# Relations between Child and Parent Fears and Changes in Family Functioning Related to COVID-19

**DOI:** 10.3390/ijerph18041786

**Published:** 2021-02-12

**Authors:** Sabrina Suffren, Karine Dubois-Comtois, Jean-Pascal Lemelin, Diane St-Laurent, Tristan Milot

**Affiliations:** 1 Département de Psychologie, Université du Québec à Trois-Rivières, Trois-Rivières, QC G9A 5H7, Canada; sabrina.suffren@uqtr.ca (S.S.); diane.st-laurent@uqtr.ca (D.S.-L.); 2 Centre de Recherche Universitaire sur les Jeunes et les Familles (CRUJeF), Québec, QC G1C 3S2, Canada; Jean-Pascal.Lemelin@usherbrooke.ca (J.-P.L.); Tristan.Milot@uqtr.ca (T.M.); 3 Centre de Recherche and Hôpital en Santé Mentale Albert-Prévost, CIUSSS du Nord-de-l’Ile-de-Montreal, Montreal, QC H4K 1B3, Canada; 4 Département de Psychoéducation, Université de Sherbrooke, Sherbrooke, QC J1K 2R1, Canada; 5 Département de Psychoéducation, Université du Québec à Trois-Rivières, Trois-Rivières, QC G8Z 4M3, Canada

**Keywords:** COVID-19, lockdown, anxiety, fear, family functioning, sleep habits, health services

## Abstract

In adults, higher anxiety level related to COVID-19 has been associated with having a pre-existing medical or mental health condition and poor sleep quality. However, no study yet has looked at these links in children. The present study’s main aim was to assess family changes associated with child and parent fears and concerns about COVID-19. We conducted a cross-sectional study among 144 families with children aged 9–12 years during the COVID-19 lockdown period. Families came from Quebec, Canada, and the survey was done in the early stages of the lockdown (April–May 2020). A phone-based survey assessed parent and child COVID-19-related fears and concerns, family-related changes and health issues. Results showed the more fears parents have about COVID-19, the more fears their child also has. Moreover, changes in family sleep habits were associated with parental and child fears and concerns about COVID-19. Reduced access to health services was associated with parental concerns about COVID-19. If another lockdown was to be put in place in the future, it would be important to inform families on the importance of sleep schedules and to maintain or increase health appointments when possible.

## 1. Introduction

Faced with the COVID-19 pandemic, most countries around the world have, at some point, established lockdowns and closed universities, schools and daycare centers, as well as all non-essential stores, to guarantee social distancing and isolation. These measures, which constituted a dramatic change in families’ habits, were particularly restrictive for parents and children who were then forced to stay at home, with some exceptions (e.g., to buy food or drugs, exercise outdoors or for essential work). Even if the measured impact of the pandemic on family well-being is still unknown [1], several studies have found higher anxiety symptoms in adults related to the outbreak and lockdown [2,3,4,5,6,7,8,9,10,11]. Adults were also worried about a friend, a family member or themselves catching COVID-19 [3]. Financial worries, loss of employment, loneliness, low social support, being a woman and COVID-19 concerns were associated with greater anxiety symptoms in adults [2,3,4,7,10,11]. 

Although parents seem to be at greater risk to develop anxiety and depression during the pandemic [2,7,11], few studies have focused on this population [12]. One preprint study has looked at potential predictors of anxiety and distress associated with quarantine in parents specifically [13]. Authors reported a link between higher anxiety and concerns about the possibility of being infected and financial impacts of the lockdown. Results also showed higher anxiety to be linked with having a pre-existing medical or mental health condition and observed distress in their children [13]. The two factors that contributed the most to anxiety were poor sleep quality and spending more time reading the news or talking about COVID-19 with family and friends [13]. Another preprint study on families showed greater parental anxiety symptoms during the pandemic, compared to pre-pandemic data [12]. In this last study, pre-existing financial deprivation, parental chronic physical or mental health condition, and COVID-19 stressors (e.g., job loss, employment changes, illness, food shortages, housing or financial insecurity) were associated with greater parental anxiety symptoms [12]. Another study reported increased physical and mental health problems in parents and children between March and June 2020 [14].

Family studies have also found greater anxiety symptoms [15,16,17,18,19,20,21,22,23,24,25] worries and fears [15,19,26], acute stress and posttraumatic stress disorders [27], as well as sleep disturbances [19] in children, related to the outbreak and lockdown. Pre-existing financial deprivation [12,25], parental chronic physical or mental health condition [12], COVID-19 stressors (e.g., job loss, employment changes, illness, food shortages, housing or financial insecurity) [12] and having relatives infected with COVID-19 [25] were associated with higher child anxiety symptoms. Additionally, being a girl [17,23,24] has been associated with higher anxiety levels during the COVID-19 outbreak [15,20]. However, we do not know if child anxiety was related to sleep disturbances or having a pre-existing medical or mental health condition. In addition, the links between anxiety levels and access to health services and child custody arrangements have not been studied, either in parents or in children. It is relevant to assess whether child fears and concerns about COVID-19 were related to family functioning during the COVID-19 lockdown, when social contacts with friends were prohibited, in children between 9 and 12 years of age. Indeed, children experience important socialization challenges as they move from the family to the peer group during middle childhood [28] and the development of child behavior problems in middle childhood continues to be related to different aspects of the family environment [29].

The first aim of the present study was to examine if parent and child fears about COVID-19 were related to each other. We also explored which concerns about COVID-19 were related to fears of COVID-19, among both parents and children. The second aim was to identify family changes (concerning family sleep habits, access to health services, income, employment, child custody arrangements) linked to greater fears and concerns about COVID-19 in both parents and children. Third, we investigated if parent and child fears and concerns about COVID-19 varied according to child sex, family income before the COVID-19 lockdown, and parent educational level. Based on previous results, we expected that changes in sleeping quality and habits would be related to higher levels of parent and child fears and concerns about COVID-19. We also expected higher levels of parent and child fears and concerns about COVID-19 to be associated with reduced access to health services, lower income, changes in parental employment, changes in child custody arrangements and more health issues. We also expected more fears and concerns about COVID-19 in girls than boys. Finally, we expected higher levels of child and parent fears and concerns about COVID-19 in low-income families and families in which parents have less education. This study was carried out in Quebec, Canada, in the early stages of the pandemic.

## 2. Materials and Methods

### 2.1. Participants

We conducted a cross-sectional study in which we administered a phone-based survey to 144 families during the pandemic: one parent and one or more children aged 9 to 12. We chose to study this age group specifically because children experience important socialization challenges during middle childhood [28] and because children below 9 years of age cannot reliably answer self-reported questions [30]. Families were recruited in Quebec, Canada, during COVID-19 lockdown in the early stages of the pandemic (between April and May 2020). The province of Quebec was the epicenter of Canada’s COVID-19 outbreak during the first wave, with the highest number of cases and deaths. In Quebec, the first person infected with COVID-19 was identified in February, 2020. From 13 March to 11 May, the government closed all universities, schools and daycares, as well as all non-essential stores, industries and businesses, to guarantee social distancing and isolation. Participants were recruited through invitations posted on social networks or sent by email by school administrations. Parents had to contact us by email to participate in the study. A research assistant then contacted the parents to explain the study, verify child age, assign them a participant number and ask them to complete the online consent form. All children between the ages of 9 and 12 who wanted to participate together with one of their biological parents who also wanted to participate were included in the study. Children and parents had to master French sufficiently to be able to understand all information and instructions and to answer questions. Once the online informed consent form was completed by parents and children, families were contacted by a trained research assistant to complete the questionnaires. The ten research assistants participated in a 3-h training conducted by the first two authors of the study prior to recruitment and they were in contact with the researchers during data collection for any remaining questions. Parents and children completed the survey separately and families were compensated with a $10 gift card for their participation. Parents were first interviewed and were then invited to lend the phone to their child and make sure the latter had privacy to complete the interview without being disturbed. Research assistants were not able to verify if all families complied with this request.

As within family effects were not the main goal of this study, and the fact of including several children from the same family decreases the power to estimate direct associations and introduces confounding and selection biases [31], we opted to include only one child per family for this study. All participating children were attributed a number. When there was more than one child per family, we selected the child with the lowest age to be included in this study. The research protocol was approved by the Université du Québec à Trois-Rivières research ethic committee (CER-20-266-10.20, approved 19 April 2020). Online informed consent was obtained from all participating parents and children before the survey was started. 

### 2.2. Measures

#### 2.2.1. COVID-19 Outbreak and Lockdown Related Fears and Concerns 

The fear of COVID-19 scale was self-assessed by parents and children separately. This scale is a unidimensional 7-item, 5-point Likert scale (1 = strongly disagree to 5 = strongly agree), measuring thoughts, behaviors and physiological symptoms related to fear of COVID-19 [32]. Example items include: “*I am afraid of losing my life because of coronavirus-19*” and “*It makes me uncomfortable to think about coronavirus-19*”. The *fear of COVID-19 scale* has been developed, used and psychometrically evaluated in adults and adolescents [32,33,34]. The *fear of COVID-19 scale* is valid and reliable, with robust psychometric properties in several languages [32,33,34]. To our knowledge, this study was the first to use this scale with children.

Parents also answered, first for themselves and also for their child, the following question related to eight different concerns about COVID-19: “*Do you or your child have concerns or worries on the following issues about COVID-19? Rate each issue (on a 1–10 scale) separately for yourself and your child*”. The first four concerns were about physical and mental health for oneself and for loved ones. The other four concerns were about: financial resources, child academic performance, family difficulties (e.g., conflicts), and access to services (e.g., physical or mental health).

#### 2.2.2. COVID-19 Outbreak and Lockdown Related Changes and Health Issues

Parents reported on six potential family changes: “Has COVID-19 brought changes in your family in the following spheres?”: family sleeping habits; access to health services (e.g., physical and mental health); family income; parental employment situation; spouse’s employment situation; child custody arrangements. Parents were also asked if any family members suffered from COVID-19 or had risk factors for COVID-19 (e.g., asthma, lung, heart or immune system problems, chronic diseases, cancer).

#### 2.2.3. Sociodemographic Variables

Parents completed a sociodemographic questionnaire that provided information on family background (family income prior to COVID-19, parents’ education, and each family members’ age and sex).

### 2.3. Statistical Analyses

Data were analyzed using SPSS v.25 (Armonk, NY, USA). There were no missing data. We used Pearson’s correlations (or Spearman’s correlations when data were not normally distributed, even after logarithmic transformations) to assess links between fears and concerns of parents and children. We used *t*-tests (or Mann-Whitney tests when data were not normally distributed) to compare fears and concerns about COVID-19 as a function of changes related to COVID-19 (presence/absence), cases of COVID-19 in the family (yes/no), cases of people with risk factors for COVID-19 in the family (yes/no) and child sex. We used Pearson correlations (or Spearman correlations when data were not normally distributed) to evaluate links between fears and concerns of parents and children, on the one hand, and continuous variables of interest (i.e., income before COVID-19, years of parental education and child age), on the other hand. Given the large number of analyses carried out, we set the alpha level at 0.01. 

Finally, we conducted multiple regression analyses predicting parental and child COVID-19 related fears and concerns, when at least two predictors were significant in previous analyses (correlations and *t*-tests). Significant predictors in previous bivariate analyses were entered simultaneously into the same regression model. 

## 3. Results

### 3.1. Sociodemographic Characteristics of Study Participants

The sample consisted of 144 children (74 boys and 70 girls) and their parent. Among the 144 responding parents, 131 were women, 13 were men. Fathers are therefore under-represented in this study. In order to ensure that results were not influenced by parental sex, analyses were first conducted by including only mothers, then including all families. The results were not different, so we carried the subsequent analyses with the complete sample. Of these 144 families, 15 (11%) earned a yearly income under 30,000$ CAN; 15 (11%) earned between 30,000$ and 49,999$ CAN; 22 (15%) earned between 50,000$ and 69,999$ CAN; 16 (11%) earned between 70,000$ and 89,999$ CAN; 29 (20%) earned between 90,000$ and 110,000$ CAN; 45 (32%) earned more than 110,000$ CAN. The average family income before COVID-19 was 98,197$ CAN. In Quebec, the median income of a two-parent family with one child was 81,810$ CAN in 2016 [35]. Thus, this sample had higher than average family income. 

### 3.2. COVID-19 Related Fear and Concerns

Parental (*M* = 14.6 ± 5.7) and child (*M* = 14.1 ± 5.7) fear levels about COVID-19 were significantly correlated (*r* = 0.26, *p* = 0.002). Parent fear of COVID-19 was significantly linked to parental concerns about: own physical (*r* = 0.53, *p* < 0.001) and mental health (*r* = 0.30, *p* < 0.001); physical health of loved ones (*r* = 0.23, *p* = 0.005); and access to health services (*r* = 0.25, *p* = 0.003). Child fear of COVID-19 was significantly linked to parent-reported child concerns about access to health services (*r* = 0.21, *p* = 0.001). 

### 3.3. COVID-19 Fear/Concerns and COVID-19 Outbreak and Lockdown Related Changes and Health Issues

#### 3.3.1. Descriptive Statistics Regarding COVID-19 Related Changes and Health Issues

Seventy percent of responding parents reported changes in their employment status (changes included home-working, stop working, fewer or more working hours); 59% reported changes in their spouse’s employment status (changes included home-working, stop working, fewer or more working hours); 13% reported changes in shared custody arrangements; 44% reported changes in family income (changes included reduced or increased income and Canadian emergency benefit or employment insurance); 69% reported changes in family sleep habits (changes included less regular sleep schedules, difficulty sleeping, insomnia, nightmares, sleeping more or less); 57% reported changes in access to health services (changes included canceled, rescheduled or phone appointments); 8% reported a family member who had COVID-19; 51% reported a family member with risk factors for COVID-19 (risk factors included asthma, hypertension, immune, chronic, cardiac or lung disease). 

#### 3.3.2. Links between COVID-19 Fear/Concerns and COVID-19 Related Changes and Health Issues 

There were no significant group differences on parent and child COVID-19 fear/concerns as a function of: changes in employment status of the responding parent and spouse; changes in shared custody arrangements; whether a member of the family had COVID-19 or not; whether a member of the family had COVID-19 risk factors or not. However, analyses revealed significant differences as a function of COVID-19 related changes in family income, sleep habits and access to health services. Significant differences are shown in Table 1. 

Parents who reported changes in family income were more concerned about financial resources. No other COVID-19 fear/concern was associated with changes in family income. Parents who reported changes in family sleep habits had more fear about COVID-19 and were more concerned about: own mental health; mental health of loved ones; financial resources; and family difficulties. Children whose parents reported changes in family sleep habits were more concerned about: own mental health; mental health of loved ones; and family difficulties. No other COVID-19 fear/concern was associated with changes in family sleep habits. Parents who reported changes in access to health services were more concerned about: loved ones’ mental health; and family difficulties (see Table 1). No other COVID-19 fear/concern was associated with changes in access to health services.

### 3.4. COVID-19 Fear/Concerns and Sociodemographic Variables 

Family income before COVID-19 was negatively correlated to parental fear about COVID-19 and parental concerns about own physical health and financial resources (See Table 1). Parent’s years of education were negatively correlated to parent fear about COVID-19 (See Table 1). There were no significant differences on COVID-19 fear/concerns as a function of child sex and age. 

### 3.5. Regression Analyses 

Multiple regressions were performed on COVID-19 fear/concerns when two or more predictors were significant in previous correlation or t-test analyses. Thus, we performed multiple regressions for parent fear of COVID-19, parent concerns about loved ones’ mental health, parent concerns about financial resources, and parent concerns about family difficulties (see Table 2). 

A multiple regression was performed to assess whether changes in family sleep habits, family income before COVID-19 and parent’s years of education, when considered together, were related to parent fear of COVID-19. A non-significant association between standardized predicted values and the absolute value of the standardized residuals showed homoscedasticity of the data [36]. Tests to see if the data met the assumption of no multicollinearity indicated that multicollinearity was not a concern (VIF of 1 and tolerance greater than 0.70 for each variable). The model was significant (*F* = 7.31, *p* < 0.001) and explained 14% of the variance (r^2^ = 0.14). Specifically, standardized beta coefficients showed that changes in family sleep habits (*β* = 0.24) and lower parent’s years of education (*β* = −0.22), but not family income before COVID-19, were predictive of parental fear about COVID-19 in the context of the COVID-19 lockdown. 

A multiple regression was conducted to assess whether changes in family sleep habits and changes in access to health services, when considered together, were related to parents’ concerns about loved ones’ mental health. A non-significant association between standardized predicted values and the absolute value of the standardized residuals showed that the data were homoscedastic. Tests to see if the data met the assumption of no multicollinearity indicated that multicollinearity was not a concern (VIF of 1 and tolerance of 0.94 for both variables). The model was significant (*F* = 11.93, *p* < 0.001) and explained 15% of the variance (*r*^2^ = 0.15). Specifically, standardized beta coefficients showed that changes in family sleep habits (*β* = 0.17) and changes in access to health services (*β* = 0.30) were both predictive of parental concerns about loved ones’ mental health in the context of the COVID-19 lockdown. 

A multiple regression was performed to assess whether changes in family income, changes in family sleep habits and family income before COVID-19, when considered together, were related to parents’ concerns about financial resources. Given heteroscedasticity of the data, regression analysis was performed using the log-transformed variables. Using these transformed variables, a non-significant association between standardized predicted values and the absolute value of the standardized residuals was obtained, showing that the log-transformed data were homoscedastic. Tests to see if the data met the assumption of no multicollinearity indicated that multicollinearity was not a concern (VIF of 1 and tolerance greater than 0.90 for each variable). The model was significant (*F* = 15.34, *p* < 0.001) and explained 25% of the variance (*r*^2^ = 0.25). Specifically, standardized beta coefficients showed that changes in family income (*β* = 0.35), changes in family sleep habits (*β* = 0.22) and lower family income before COVID-19 (*β* = −0.22) were all predictive of parental concerns about financial resources in the context of the COVID-19 lockdown.

A multiple regression was conducted to assess whether changes in family sleep habits and changes in access to health services, when considered together, were related to parents’ concerns about family difficulties. Given the heteroscedasticity of the data, regression analysis was performed using the log-transformed variables. Using these transformed variables, a non-significant association between standardized predicted values and the absolute value of the standardized residuals was obtained, showing that the log-transformed data were homoscedastic. Tests to see if the data met the assumption of no multicollinearity indicated that multicollinearity was not a concern (VIF of 1 and tolerance of 0.94 for both variables). The model was significant (*F* = 16.69, *p* < 0.001) and explained 19% of the variance (*r*^2^ = 0.19). Specifically, standardized beta coefficients showed that changes in family sleep habits (*β* = 0.37) and changes in access to health services (*β* = 0.16) were both predictive of parental concerns about family difficulties in the context of the COVID-19 lockdown.

## 4. Discussion

Studies have shown that the COVID-19 outbreak and lockdown was associated with increased anxiety, fears and concerns about COVID-19 in families and children [15]. We studied 144 families with children aged between 9 and 12 in order to assess links between fears/concerns about COVID-19 and changes experienced by families during the COVID-19 outbreak and lockdown. Our study highlights associations between changes in family sleep habits and parental and child COVID-19 related fears/concerns. Reduced access to health services was also linked to parental concerns, but not to parent-reported child concerns. 

Our results showed that parent fear about COVID-19 (self-reported) was significantly related to child fear about COVID-19 (self-reported). The more fears parents have about COVID-19, the more fears of COVID-19 their child also has. This result can be explained in different ways. In fact, anxiety, fears and concerns can be transmitted from parent to child, either through genetic mechanisms or through the environment. Environmental factors that may explain intergenerational transmission of anxiety, fears and concerns include parenting, parent-child relationships, parental stress, or the child’s characteristics (e.g., temperament or cognitive abilities) [37,38,39]. Moreover, this is a bidirectional effect and greater levels of child anxiety, fears and concerns can also be linked with greater levels of parental anxiety, fears and concerns [37,38,39]. Anxiety, fears and concerns were potentially amplified by the pandemic stressful context. In the face of fears and concerns about COVID-19, clinicians should target all family members in order to reduce child fears and concerns. Our findings also revealed that fear specific to COVID-19 was linked to several non-specific (contextual) concerns about COVID-19 among parents (self-reported), and to a lesser extent, among children (parent-reported). These results show that parents are not only afraid of catching the disease, but are also anxious about many aspects of the life context associated with the COVID-19 pandemic and its social and economic effects. The fact that associations between fear of COVID-19 and contextual concerns about COVID-19 were both fewer and weaker in children may have to do with child contextual concerns being reported by parents, while specific fear of COVID-19 was reported by the children themselves. It is therefore likely that parent-reported child contextual concerns were influenced in part by parental own specific fears and contextual concerns [20]. Future studies should also question children about their own concerns.

Results with regard to sleep habits suggest that the quality of sleep appears particularly important for the prevention of fears and concerns about COVID-19 in children and parents during lockdown. These results are in accordance with those from several other studies, both in adults and children, which showed the importance of the link between sleep quality and anxiety level [13,40,41], and between dreams (i.e., heightened dream recall, more negatively toned dreams, higher emotional dream intensity, pandemic-related dreams) and anxiety level at the time of COVID-19 [42,43,44]. These studies promoted good sleep quality in a potentially stress generating context such as the COVID-19 pandemic [13,40,41]. Solutions proposed to promote a good quality of sleep were to keep a regular sleep schedule [13,41], exercise regularly [41], use one’s bed only for sleep [13], and not use electronic devices immediately prior to bed (30–60 min) [13]. This last recommendation was linked to not pay too much attention to COVID-19 outbreak information before going to sleep [41]. Although it is impossible to know from the present study whether fears and concerns about COVID-19 generated sleep problems or if sleep problems led to fears and concerns about COVID-19, this relation is most probably bidirectional, particularly among children and adolescents [40,45,46]. 

Another important result from our study was the increased parental concerns associated with canceled or reported health appointments (physical or mental health, developmental follow-ups), which were related to concerns with mental health of loved ones and family difficulties. The results supports studies showing a beneficial effect of telehealth follow-ups during the lockdown, particularly with regard to mental health [47]. Studies should now focus on the use of health services following the lockdown. Indeed, it is likely that health services use will increase drastically after a significant interruption of services (i.e., several months), which could result in difficulties in getting appointments promptly and increase anxiety even more. The consequences of health services interruptions during lockdown are likely to have longer-term consequences for families. In order to limit these consequences, the services offered by the *health* and *social services providers* *should be maintained and even increased in the next months*, particularly in the perspective of a second or even third wave of COVID-19. In addition, health and social services providers should now think about mechanisms to limit decreased services in the case of re-lockdown and to better support the families that are more vulnerable or anxious due to COVID-19.

A lower family income before COVID-19 was linked to higher parental fears about COVID-19 and concerns about financial resources. A lower level of parental education was also linked to higher parental fears about COVID-19. The association between family income before COVID-19 and parental fears was, however, nonsignificant when changes in family sleep habits and parent education were also considered in the model. Taken together, these results suggest that lower-income families and those with less educated parents are at higher risk of presenting fears and concerns related to COVID-19. They could represent a group at risk requiring more attention from health and social services workers.

Results from the present study did not show differences between girls and boys about fears and concerns. These results are different from those of previous research showing that girls and women were more likely to show anxiety than boys and men [17,23,48,49]. These results suggest that fears related specifically to COVID-19 and contextual concerns about COVID-19 do not vary much as a function of sex. Interventions targeting the reduction of fears and concerns about COVID-19 in children should therefore be similar for girls and boys.

Results from the current study provided information on families’ fears and concerns about COVID-19 during the first weeks of lockdown in Quebec, Canada. However, we know nothing about participants’ fears and concerns prior to the COVID-19 pandemic. These results should therefore be interpreted with caution. In addition, it is important to keep in mind that we cannot test or infer causality here, based on the correlational and cross-sectional design of this study. Another limitation of this study is that most of the measurements were completed by the parent and not by the child. As a result, it is possible that we missed some effects of lockdown on children, and that the parent’s perspective does not fully reflect the child’s reality, particularly with regard to child concerns. Indeed, parental symptoms can bias parents’ ratings of their child’s symptoms [50]. Moreover, only one parent answered questions about child concerns and the shared method variance may have inflated some of the observed links. Future studies should favor a multi-method approach (e.g., both parents, children and teacher reports, and observations). On the other hand, it is also possible that the responding parent’s sex influenced the results. Indeed, among the 144 families interviewed, 131 respondents were mothers and 13 were fathers. Even if the results were similar whether fathers were included or not, we do not know if the fathers’ perspective regarding changes related to lockdown and child concerns would have been different from the mothers’. Additionally, we do not know fathers’ fears and concerns about COVID-19 levels. Future studies should carry out the measurements in both mothers and fathers independently. In addition, although adequate to test study hypotheses, the number of participants was limited when compared to cohort studies. Moreover, given the large number of analyses performed, it is possible that some significant results at *p* < 0.01 are due to chance. Finally, the *fear of COVID-19 scale* has been developed, used and psychometrically evaluated only in adults and adolescents. Indeed, to our knowledge, this study was the first to use this scale with children. Since child fear of COVID-19 was linked to parent fear of COVID-19 and various contextual factors related to lockdown, this scale appears suitable for use with children 9 to 12 years old. 

## 5. Conclusions

The COVID-19 outbreak and lockdown has had a clear impact on parents’ and children’s well-being, fears and concerns. If another lockdown were to be put in place in the future, specific needs of families with children should be taken into account. One recommendation would be to inform families on the importance of regular sleep schedules and healthy sleep habits. It would also be important to try to maintain health appointments through the duration of the lockdown. It is very likely that many children will develop anxiety disorders in the coming months or years in relation to their experience of the COVID-19 outbreak and lockdown. Intervening as early as possible with families could allow interventions to be put in place before children develop more chronic disorders.

## Figures and Tables

**Table 1:** COVID-19 fear/concerns in association with COVID-19 related changes and sociodemographic variables: Significant results.

	Changes Related to COVID-19			Sociodemographic Variables	
	Family Income	Family Sleep Habits	Access to Health Services	Family Income before COVID-19	Parent’s Years of Education
	*t*-test	*t*-test	*t*-test	R	r
Parents’ fear and concerns					
Fear of COVID-19 scale		−3.36 *		−0.28 *	−0.28 *
Their physical health				−0.24 *	
Their mental health		−4.15 **			
Loved ones’ physical health					
Loved ones’ mental health		−2.95 *	−4.38 **		
Financial resources	−4.77 **	−3.36 *		−0.27 *	
Child’s academic performance					
Family difficulties		−5.32 **	−3.12 *		
Access to health services					
Children’s fear and concerns					
Fear of COVID-19 scale					
Their physical health					
Their mental health		−3.18 *			
Loved ones’ physical health					
Loved ones’ mental health		−2.57 *			
Financial resources					
Their academic performance					
Family difficulties		−3.99 **			
Access to health services					

Note. * *p* < 0.01; ** *p* < 0.001.

**Table 2:** Multiple regressions testing associations between parents’ COVID-19 fear/concerns and lockdown related changes and health issues.

.	*B*	*SE*	*β*	*p*	*95%* CI
Parents’ fear of COVID-19					
Changes in family sleep habits	2.97	0.99	0.24	0.003	1.00, 4.93
Family income before COVID-19					
Parent’s years of education	−0.35	0.15	−0.22	0.021	−0.64, −0.05
Parents’ concerns about loved ones’ mental health					
Changes in family sleep habits	0.91	0.44	0.17	0.041	0.04, 1.78
Changes in access to health services	1.55	0.41	0.30	<0.001	0.74, 2.36
Parents’ concerns about financial resources					
Changes in family income	0.24	0.05	0.35	<0.001	0.14, 0.35
Changes in family sleep habits	2.02	0.70	0.22	0.005	0.63, 3.41
Family income before COVID-19	−0.23	0.08	−0.22	0.004	−0.39, −0.08
Parents’ concerns about family difficulties					
Changes in family sleep habits	3.40	0.72	0.37	<0.001	1.97, 4.83
Changes in access to health services	0.11	0.05	0.16	0.038	0.01, 0.22

## Data Availability

The data presented in this study are available on request from the corresponding author. The data are not publicly available due to being unnecessary for the understanding of the document.
